# Frailty is a risk factor for occupational falls among older workers: an internet-based prospective cohort study

**DOI:** 10.1093/joccuh/uiae065

**Published:** 2024-10-28

**Authors:** Ryutaro Matsugaki, Yoshihisa Fujino, Masayoshi Zaitsu, Satoru Saeki, Shinya Matsuda, Akira Ogami

**Affiliations:** Department of Work Systems and Health, Institute of Industrial Ecological Sciences, University of Occupational and Environmental Health, 1-1, Iseigaoka, Yahatanishiku, Kitakyushu 807-8555, Japan; Department of Environmental Epidemiology, Institute of Industrial Ecological Sciences, University of Occupational and Environmental Health, 1-1, Iseigaoka, Yahatanishiku, Kitakyushu 807-8555, Japan; Center for Research of the Aging Workforce, University of Occupational and Environmental Health, 1-1, Iseigaoka, Yahatanishiku, Kitakyushu 807-8555, Japan; Department of Rehabilitation Medicine, School of Medicine, University of Occupational and Environmental Health, 1-1, Iseigaoka, Yahatanishiku, Kitakyushu 807-8555, Japan; Department of Preventive Medicine and Community Health, School of Medicine, University of Occupational and Environmental Health, 1-1, Iseigaoka, Yahatanishiku, Kitakyushu 807-8555, Japan; Department of Work Systems and Health, Institute of Industrial Ecological Sciences, University of Occupational and Environmental Health, 1-1, Iseigaoka, Yahatanishiku, Kitakyushu 807-8555, Japan

**Keywords:** frailty, occupational fall, older worker, risk factor

## Abstract

Objectives: Occupational falls are a significant concern among older workers. Although recent cross-sectional studies have indicated a potential association between frailty and occupational falls among older workers, the causal relationship remains unclear. This longitudinal study aimed to investigate whether frailty is a risk factor for occupational falls among older workers using a longitudinal design.

Methods: This was an internet-based prospective cohort study. A total of 5000 older workers (aged 60-75 years) were recruited, with 2873 participants meeting the inclusion criteria for the follow-up survey. Frailty was assessed using a frailty screening index based on the Fried phenotype model. Occupational falls were defined as those that occurred during the follow-up period.

Results: Among the participants, 13.9% were frail. The incidence of occupational falls was higher in the frailty group (11.6%) than in the nonfrailty group (4.9%). In the multivariate-adjusted model, frailty was significantly associated with occupational falls (relative risk: 2.10; 95% CI, 1.51-2.94).

Conclusions: Frailty is a significant risk factor for occupational falls among older workers. Employers should consider implementing health-management strategies that focus on frailty to prevent occupational falls in this population.

## Teaser

Key points

What is already known on this topic: Among older workers, falls are a significant cause of occupational injuries, with frailty being suggested as a potential risk factor. However, longitudinal studies have not established the causal relationship between frailty and occupational falls.

What this study adds: This longitudinal study provides evidence that frailty is significantly associated with an increased risk of occupational falls among older workers. It highlights that specific components of frailty, such as weight loss, low physical function, and exhaustion, are particularly associated with fall risk.

How this study might affect research, practice, or policy: The study findings underscore the need for employers to implement health management strategies targeting frailty to prevent occupational falls. Future research should focus on developing and testing interventions aimed at reducing frailty to enhance workplace safety for older workers.

## 1. Introduction

Falls are among the most common causes of occupational injuries, accounting for approximately 20%-30% of all work-related accidents.^1^^‑^^3^ Falls are associated with health-related work limitations^4^ and contribute to approximately 20% of work-related hospitalizations,^5^ highlighting their significant impact not only on individual workers but also on society. Occupational falls are closely related to aging, with the incidence peaking around the age of 60 years.^5^^,^^6^ The employment rate of older workers is increasing, particularly in developed countries,^7^^‑^^9^ and the issue of occupational falls among older workers is likely to become more pronounced in the future. Therefore, developing effective strategies to prevent occupational falls among older workers is an urgent occupational health issue. Identifying the risk factors of occupational falls in this population is essential.

Frailty is associated with falls in older workers.^10^^,^^11^ It is characterized by a decline in physiological reserves and increased vulnerability to stress and is recognized as a public health issue.^12^^‑^^14^ Our previous cross-sectional study revealed that frailty is associated with occupational falls among older workers aged ≥60 years (median age: 63 years).^11^ Similar findings were reported in a cross-sectional study involving older workers with a mean age of 75 years.^10^ These results highlight the need to focus on frailty as a preventive strategy against occupational falls among older workers.

However, to our knowledge, no longitudinal study has examined the association between frailty and occupational falls in older workers. Falls can induce fear of falling, which is known to increase the risk of frailty.^15^ This finding suggests that falls can be considered a risk factor for frailty. Therefore, it is necessary to use longitudinal data to determine whether frailty is a risk factor for occupational falls among older workers.

This longitudinal study aimed to investigate whether frailty is a risk factor for occupational falls among older workers. We hypothesized that frailty is a risk factor for occupational falls among older workers.

## 2. Methods

This prospective cohort study used data from an internet survey to comprehensively examine the relationship between socioeconomic status, health status, occupational injuries, work-related disabilities, and health among older workers in the tertiary sector. This study was approved by the Ethics Committee of Medical Research at the University of Occupational and Environmental Health, Japan (approval number: R4-031). Informed consent was obtained from all respondents using a checkbox provided on the questionnaire response screen.

The baseline survey was conducted in September 2022.^11^ Specifically, we sent questionnaires to individuals registered as employed at the time of registration in a survey panel managed by Cross Marketing Inc (Tokyo). The exclusion criteria were as follows: (1) unemployed at the time of the survey, (2) age <60 or >75 years, (3) self-employed or unpaid family workers in a family business, (4) not employed in the tertiary sector, and (5) provided invalid responses on the questionnaire. The survey was closed after collecting responses from 5000 participants who did not meet any exclusion criteria. Sampling was conducted to ensure a 1:1 sex ratio. Follow-up surveys were conducted in October 2023 with the 5000 participants who provided data in the baseline survey. Responses were received from 3113 participants. After excluding invalid responses (*n* = 240), 2873 participants were included in the study.

### 2.1. Assessment of frailty

Frailty was assessed using a frailty screening index (FSI) based on the Fried phenotype model.^16^^,^^17^ The FSI evaluates frailty primarily from a physical perspective (physical frailty).^18^ The FSI comprises 5 questions addressing weight loss, low physical activity, low physical function, cognitive decline, and exhaustion. Participants responded to these questions with either a "yes" or "no." In this study, frailty was characterized by the presence of at least 3 out of 5 specific criteria: weight loss, low physical activity, low physical function, cognitive decline, and exhaustion.^17^

### 2.2. Assessment of occupational falls

We investigated the experience of occupational falls using an approach similar to that of a previous study,^11^ and defined occupational falls during the follow-up period as follows: having 1 or more occupational falls as "at least one occupational fall," having 2 or more occupational falls as "recurrent occupational falls," and having a fall that resulted in an injury requiring medical attention as "occupational fall with injury.” In this study, we defined a fall as an unexpected event in which the participant comes to rest on the ground, floor, or a lower level.^19^^,^^20^ In addition, we expanded this definition to include any unintentional contact of any part of the body with the ground, floor, or lower level.

### 2.3. Assessment of covariates

We examined the following variables at baseline as covariates: age, sex, educational background, subjective economic status, medical condition, medication, employment status, job description, primary work location, frequency of work per week, working hours per day, industry, and company size.

To assess the subjective economic status, we asked the participants to rate their current living situation from an economic perspective. We collected responses using a 5-point Likert scale (very comfortable, somewhat comfortable, normal, somewhat difficult, or very difficult). Participants who selected "somewhat difficult" or "very difficult" were classified as “experiencing economic difficulty.”

For medications, we examined polypharmacy and fall-risk-increasing medications to be associated with falls.^21^^‑^^23^ We defined polypharmacy as the use of ≥5 medications with reference to previous studies.^21^^,^^22^ In this study, fall-risk-increasing medication use was defined as the use of 1 or more hypnotics, antiepileptics, antipsychotics, anxiolytics, or antidepressants.^23^^‑^^25^ Regarding medical conditions, we examined diabetes and hypertension, which have been associated with occupational falls in previous studies.^23^

### 2.4. Statistical analysis

We performed a modified Poisson regression analysis (Poisson regression with a robust error variance)^26^ with occupational falls as the dependent variable and frailty status as the explanatory variable to obtain the relative risk (RR) and 95% CI. First, we performed an analysis adjusted for age and sex (age- and sex-adjusted model). Additionally, we calculated the RRs for age, sex, educational level, subjective economic status, medical condition, medication, employment status, job description, primary work location, frequency of work per week, working hours per day, industry, and company size (multivariate-adjusted model). Moreover, the relationship between the frailty components and occupational falls was examined using a multivariate-adjusted model.

Stata MP/18.0 (StataCorp LLC, TX, USA) was used for statistical analyses, with significance level set at 5%.

### 2.5. Sensitivity analysis

We conducted a sensitivity analysis considering exposure misclassification. We defined frailty as an FSI score ≥3. However, previous research suggests that defining frailty as an FSI score ≥2 is more appropriate.^16^ When frailty is defined as an FSI score ≥3, there is a possibility of nondifferential misclassification, which may result in the underestimation of the association between frailty and occupational falls. To address this issue, we redefined frailty as an FSI score ≥2 and examined the association between frailty and occupational falls using the same analytical methods as in the primary analysis.

## 3. Results

Table 1 shows the characteristics of the subjects. Among the 2873 participants, the prevalence of frailty was 13.9% (398/2873). Compared with the nonfrail group, the frail group had a higher proportion of those reporting difficult subjective economic status (28.6% vs 54.0%), polypharmacy (11.1% vs 22.9%), and the use of fall-risk-increasing drugs (6.3% vs 15.1%).

**Table 1:** Characteristics of participants.

	** Total (*n* = 2873)**	**Frailty**	
		**Without (*n* = 2475)**	**With (*n* = 398)**
**Age, median (interquartile range), y**	63.0 (61.0, 66.0)	63.0 (61.0, 66.0)	62.0 (61.0, 65.0)
**Male sex, *n* (%)**	1605 (55.9)	1373 (55.5)	232 (58.3)
**Educational background, *n* (%)**			
**Junior high school/high school**	764 (26.6)	645 (26.1)	119 (29.9)
**Vocational school/college**	575 (20.0)	490 (19.8)	85 (21.4)
** University**	1534 (53.4)	1340 (54.1)	194 (48.7)
**Subjective economic status (difficult), *n* (%)**	922 (32.1)	707 (28.6)	215 (54.0)
**Medical condition, *n* (%)**			
**Diabetes**	255 (8.9)	203 (8.2)	52 (13.1)
**Hypertension**	941 (32.8)	781 (31.6)	160 (40.2)
**Medication use**			
**Polypharmacy**	365 (12.7)	274 (11.1)	91 (22.9)
**Fall-risk-increasing drug**	217 (7.6)	157 (6.3)	60 (15.1)
**Employment status, *n* (%)**			
**Regular employment**	1205 (41.9)	1026 (41.5)	179 (45.0)
**Nonregular employment**	1668 (58.1)	1449 (58.5)	219 (55.0)
**Job description, *n* (%)**			
**Not mainly manual work**	2038 (70.9)	1753 (70.8)	285 (71.6)
**Mainly manual work**	835 (29.1)	722 (29.2)	113 (28.4)
**Primary work location, *n* (%)**			
**Outdoor**	355 (12.4)	298 (12.0)	57 (14.3)
**Indoor**	2518 (87.6)	2177 (88.0)	341 (85.7)
**Work frequency, *n* (%), d/wk**			
**<3**	212 (7.4)	190 (7.7)	22 (5.5)
**3-4**	712 (24.8)	625 (25.3)	87 (21.9)
**≥5**	1949 (67.8)	1660 (67.1)	289 (72.6)
**Working hours, *n* (%), h/d**			
**<3**	47 (1.6)	43 (1.7)	4 (1.0)
**3-4**	573 (19.9)	505 (20.4)	68 (17.1)
**≥5**	2253 (78.4)	1927 (77.9)	326 (81.9)
**Industry, *n* (%)**			
**Wholesale, retail**	690 (24.0)	584 (23.6)	106 (26.6)
**Medical, health care and welfare**	538 (18.7)	468 (18.9)	70 (17.6)
**Others**	1645 (57.3)	1423 (57.5)	222 (55.8)
**Company size (persons), *n* (%)**			
**<9**	412 (14.3)	353 (14.3)	59 (14.8)
**10-99**	935 (32.5)	802 (32.4)	133 (33.4)
**100-999**	1336 (46.5)	1165 (47.1)	171 (43.0)
**1000+**	190 (6.6)	155 (6.3)	35 (8.8)

The relationship between frailty and occupational falls is presented in Table 2. The incidence of at least 1 occupational fall was higher in the frailty group than in the nonfrailty group (11.6% vs 4.9%). In the multivariate-adjusted model, frailty was associated with at least 1 occupational fall (RR: 2.10; 95% CI, 1.51-2.94). Additionally, frailty was associated with recurrent occupational falls (RR: 2.28; 95% CI, 1.17-4.45) and occupational falls with injury (RR: 2.12; 95% CI, 1.01-4.46). The analysis, which defined frailty as an FSI score ≥2, showed similar results regarding the association between frailty and occupational falls (Table S1).

**Table 2:** Association between frailty and incidence of occupational falls.

	**Incidence of occupational falls**	**Age/sex-adjusted model**				**Multivariate-adjusted model** ^a^			
		**RR**	**95% CI**		***P* value**	**RR**	**95% CI**		***P* value**
**At least 1 occupational fall**									
**Without frailty**	4.9% (120/2475)	Reference				Reference			
**With frailty**	11.6% (46/398)	2.42	1.75	3.34	<.001	2.10	1.51	2.94	<.001
**Recurrent occupational falls**									
**Without frailty**	1.2% (29/2475)	Reference				Reference			
**With frailty**	3.5% (14/398)	2.92	1.54	5.55	.001	2.28	1.17	4.45	.015
**Occupational fall with injury**									
**Without frailty**	1.1% (28/2475)	Reference				Reference			
**With frailty**	3.0% (12/398)	2.72	1.39	5.33	.004	2.12	1.01	4.46	.047

Abbreviation: RR, relative risk.

a Multivariate-adjusted model: adjusted for age, sex, educational back ground, subjective economic status, medical condition, medication use, employment status, job description, primary work location, work frequency, working hours, industry, and company size.

**Figure 1:**
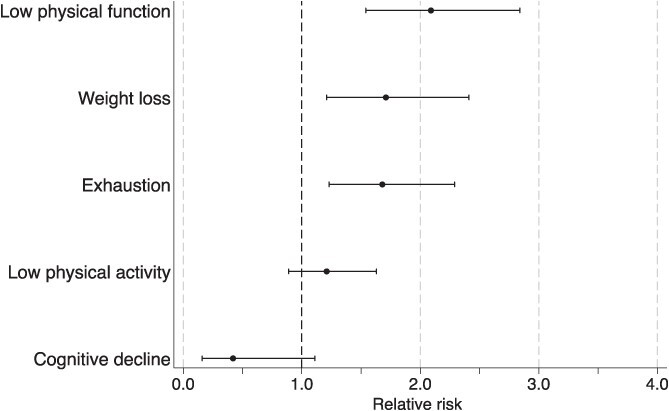
Relative risks (RRs) and 95% CIs of frailty components for the incidence of at least 1 occupational fall. RRs were estimated using a modified Poisson regression analysis adjusted for age, sex, educational level, subjective economic status, medical condition, medication, employment status, job description, primary work location, frequency of work per week, working hours per day, industry, and company size.

Figure 1 illustrates the relationship between the frailty components and at least 1 occupational fall. In the multivariate-adjusted model, low physical function (RR: 2.09; 95% CI, 1.54-2.84), weight loss (RR: 1.71; 95% CI, 1.21-2.41), and exhaustion (RR: 1.68; 95% CI, 1.23-2.29) were associated with occupational falls (Table S2).

## 4. Discussion

This study used a longitudinal design to investigate the relationship between frailty and occupational falls in older workers. The results revealed a significant association between frailty and the occurrence of occupational falls among older workers. This relationship remained robust, even after the sensitivity analyses using different definitions of frailty were conducted. Notably, this study is to our knowledge the first to reveal an association between frailty and the occurrence of occupational falls among older workers. These findings highlight the necessity of implementing preventive measures focusing on frailty to reduce occupational falls in this population.

The results of our cohort study corroborate the findings of previous cross-sectional studies that have reported an association between frailty and occupational falls among older workers.^10^^,^^11^ In studies involving community-dwelling older adults, robust evidence indicates that frailty is a risk factor for falls.^27^^‑^^29^ A recent meta-analysis of 29 cohort studies involving individuals aged ≥65 years reported that frailty is a significant risk factor for falls.^29^ Our findings suggest that the knowledge accumulated in the field of geriatrics can be applied to the understanding and prevention of occupational falls among older workers.

Among frailty components, weight loss, low physical function, and exhaustion were closely associated with occupational falls. Our findings highlight the importance of managing frailty as a preventive measure against falls in older workers. Specifically, our study emphasized the necessity of multidimensional health management, focusing on lifestyle interventions such as exercise, nutrition, and fatigue management. Conversely, cognitive decline was associated with a reduced risk of falls; however, this association was not statistically significant. This result contrasts with previous reports suggesting that cognitive decline increases the risk of falls. One possible hypothesis is that workers with cognitive decline may be assigned safer workplaces or tasks to mitigate the risk of falling. Additionally, there may be an underreporting of falls among workers with cognitive decline. Further investigations are necessary to clarify the relationship between cognitive decline and occupational falls.

The strength of our study lies in its demonstration that frailty, as measured using a simple self-administered questionnaire, can effectively identify the risk of occupational falls. Although various tools to measure frailty exist, some require direct measurement^30^^‑^^32^ and others, even if self-administered, may involve numerous items.^33^^,^^34^ Generally, assessments requiring direct measurements are often avoided in occupational health settings because of the necessity of manpower and time. Moreover, in countries such as Japan, where comprehensive health examination questionnaires are already in place,^35^ adding numerous questions for frailty assessment may not be desirable. In this regard, our study provides valuable insights into occupational health practices by suggesting that frailty, assessed using the FSI, a 5-item questionnaire with yes/no responses, can be used for risk identification.

This study had several limitations. First, the outcomes of occupational falls were assessed based on self-reported fall experiences over the past year, which may have introduced recall bias. Particularly, participants with cognitive decline may underreport fall experiences, potentially leading to an underestimation of the relationship between frailty and occupational falls. Second, our study did not account for the visual factors that have been suggested to be associated with occupational falls among older workers.^23^^,^^36^ However, the effect of this omission on our results is unclear. Falls related to visual impairment are mainly due to poor contrast sensitivity rather than near- or distance-related visual acuity.^37^ Given that the prevalence of contrast sensitivity impairment is approximately 3.7% among individuals aged 71-74 years,^38^ its impact on our study is likely to be minimal. Finally, our study focused on workers in tertiary industries, and it is uncertain whether the findings can be generalized to workers in primary and secondary industries. Nonetheless, our findings suggest that frailty is associated with occupational falls, considering job tasks and environments. Therefore, it may be possible to extrapolate our results to workers in the primary and secondary industries.

In conclusion, this study revealed that frailty is a risk factor for occupational falls among older workers. Employers of older workers should consider implementing health management strategies that focus on frailty to prevent occupational falls in this population.

## Supplementary Material

Web_Material_uiae065

## Data Availability

The data that support the findings of this study are available from the corresponding author upon reasonable request.
