# Supplementary material for: Frailty is a risk factor for occupational falls among older workers: an internet-based prospective cohort study
Source: J Occup Health. 2024 Oct 28;66(1):uiae065. doi: 10.1093/joccuh/uiae065 (PMC11635635; doi:10.1093/joccuh/uiae065)
Supplement: Web_Material_uiae065 [file web_material_uiae065.zip › Table_S1.pdf]

Table S1. Association between frailty and the incidence of occupational falls

	Incidence of occupational fall	Age–sex adjusted model				Multivariate adjusted model			
		RR	95%CI		p-value	RR	95%CI		p-value
At least one occupational fall									
Without frailty	3.6% (63/1739)	Reference				Reference			
With frailty	9.1% (103/1134)	2.51	1.86	3.41	<0.001	2.24	1.65	3.05	<0.001
Recurrent occupational fall									
Without frailty	0.6% (11/1739)	Reference				Reference			
With frailty	2.8% (32/1134)	4.43	2.24	8.76	0.001	3.64	1.81	7.32	<0.001
Occupational fall with injury									
Without frailty	0.8% (13/1739)	Reference				Reference			
With frailty	2.4% (27/1134)	3.20	1.66	6.18	0.001	2.73	1.34	5.55	0.006

Multivariate adjusted model: adjusted for age, sex, educational background, subjective economic status, medical condition, medication use, employment status, job description, primary work location, work frequency, working hours, industry, and company size.

RR: relative risk, CI: confidence interval
