# Supplementary material for: Frailty is a risk factor for occupational falls among older workers: an internet-based prospective cohort study
Source: J Occup Health. 2024 Oct 28;66(1):uiae065. doi: 10.1093/joccuh/uiae065 (PMC11635635; doi:10.1093/joccuh/uiae065)
Supplement: Web_Material_uiae065 [file web_material_uiae065.zip › Table_S2.pdf]

Table S2. Association between frailty components and the incidence of at least one occupational fall.

	Incidence of at least one occupational fall	Age–sex adjusted model				Multivariate adjusted model			
		RR	95%CI		p-value	RR	95%CI		p-value
Frailty subcomponent									
Weight loss	9.4% (38/404)	1.84	1.30	2.61	0.001	1.71	1.21	2.41	0.002
Low physical function	8.7% (98/1132)	2.24	1.65	3.03	<0.001	2.09	1.54	2.84	<0.001
Low physical activity	6.5% (94/1438)	1.31	0.97	1.76	0.076	1.21	0.89	1.63	0.222
Cognitive decline	2.8% (4/145)	0.47	0.18	1.25	0.131	0.42	0.16	1.11	0.080
Exhaustion	9.1% (61/668)	1.93	1.43	2.62	<0.001	1.68	1.23	2.29	0.001

Multivariate adjusted model: adjusted for age, sex, educational background, subjective economic status, medical condition, medication use, employment status, job description, primary work location, work frequency, working hours, industry, and company size.

RR: relative risk, CI: confidence interval.
